# Promoting appetitive learning of consensual, empowered vulnerability: a contextual behavioral conceptualization of intimacy

**DOI:** 10.3389/fpsyg.2023.1200452

**Published:** 2023-08-09

**Authors:** Louisiana Contextual Science Research Group, Jade Campbell, Jessica Criddle, LaGriff Griffin, Eva Lieberman, Michael May, Melissa Miller, Nicole Pyke, MaKensey Sanders, Emily Sandoz, Thomas Sease, Janani Vaidya, Jon-Patric Veal, Abbey Warren

**Affiliations:** University of Louisiana at Lafayette, Lafayette, LA, United States

**Keywords:** intimacy, vulnerability, consent, power, well-being, appetitive, context, behavioral

## Abstract

Vulnerability is emphasized in a number of theoretical models of intimacy (e.g., Intimacy Process Model), including from behavioral and contextual behavioral perspectives. Vulnerability is generally defined as susceptibility to harm and involves behaviors that have been historically met with aversive social consequences. From these perspectives, intimacy is fostered when vulnerable behavior is met with reinforcement. For example, interventions have trained intimacy by building skills in emotional expression and responsiveness with promising results. Vulnerability has divergent functions, however, depending on the interpersonal context in which it occurs. Functional intimacy is explored through the lens of functional relations, which play a key role in interpersonal processes of power, privilege, and consent. This conceptualization suggests that vulnerability must be under appetitive functional relations, consensual, and empowered for safe intimacy to emerge. The responsibility to promote appetitive learning of consensual, empowered vulnerability to foster intimacy falls to the person with more power in a particular interaction and relationship. Recommendations are offered for guiding this process.

## 1. Introduction

Intimacy has long been considered a fundamental aspect of human well-being and development (e.g., Erikson, 1950, 1963), and remains a key social factor in modern scientific explorations of well-being. In children, friendship intimacy buffers the relationship between symptoms of attention deficit hyperactivity disorder (ADHD) and social problems such as rejection by peers, emotional regulation, and social reciprocity (Becker et al., 2013). Naturally occurring increases in physical intimacy predict concurrent and subsequent decreases in somatic symptoms for people in romantic relationships (Stadler et al., 2012). Intimacy also mediates the positive effects of decreased loneliness and increased happiness associated with social media use (Pittman, 2018). At the societal level, overall experiences of intimacy attenuate the impact of negative outgroup experiences on attitudes toward that outgroup (Graf et al., 2020). In short, intimacy is considered a hallmark of both relational and personal well-being, despite the homogeneity of sample populations in the research (Williamson et al., 2022).

The English words “intimacy” and “intimate” are derived from Latin roots, *intimus* (innermost) and *intimare* (to make innermost known; Partridge, 2006). By literal definition, intimacy is “the state of being intimate; something of a personal or private nature” (Merriam-Webster, n.d.). In the behavioral sciences, several conceptual models of intimacy have emerged (e.g., Waring, 1985; Reis and Shaver, 1988; Wilhelm and Parker, 1988; Register and Henley, 1992; Prager, 1997, Gaia, 2002), each of which vary slightly on common themes. These models converge on defining *intimacy* as dynamic, contextually-bound (see Gaia, 2002), and involving the disclosure of thoughts, feelings, and personal information with reciprocal trust and emotional closeness (see Timmerman, 2009). In other words, historical accounts of intimacy emphasize a dynamic interpersonal process of reciprocal vulnerability.

The role of reciprocal vulnerability is seen explicitly in behavioral and contextual behavioral models of intimacy, which emphasize intimacy as the product of interactions in which vulnerable behaviors are reinforced by one’s partner’s responsiveness (Cordova and Scott, 2001). Likewise, a contextual behavioral reformulation of the Interpersonal Process Model (IPM; Reis and Shaver, 1988) posits the evolution of intimate relating as involving vulnerability being met with reinforcing responsiveness, thereby increasing the likelihood of vulnerable behaviors being emitted in the future (Kanter et al., 2020). Thus, intimacy emerges when an interaction evokes and reinforces bidirectional vulnerability.

## 2. Vulnerability

The English word “vulnerability” is derived from the Latin roots, *vulnus* (wound), and *habilitatem* (ability or capacity; Partridge, 2006). Defined literally, to be vulnerable is to engage in behavior that results in an increased capability “of being physically or emotionally wounded” (Merriam-Webster, n.d.). In other words, vulnerability colloquially involves socially risky behavior. Research on vulnerability typically revolves around describing populations that are at risk of being taken advantage of (e.g., Msall et al., 1998) and considering individual differences in emotional responding (e.g., Timmers et al., 2003). Vulnerability is increasingly being explored, however, as an aspect of well-being rather than a threat. For example, social worker, speaker, and author Brown (2013) stated that vulnerability entails “uncertainty, risk, and emotional exposure,” and is understood as necessary for personal growth and well-being. Recognizing and accepting personal vulnerability, or an “openness to attack,” is seen as a critical aspect of *shame resilience* (Brown, 2006). A similar definition of emotional vulnerability as an “aversive state” of openness to feeling hurt or rejected can be found in Vogel et al. (2003). Vulnerability, particular to the relational context, has been observed as fear of abandonment emerges in some relationships but not others (e.g., with real or potential threats of rejection; Fowler and Dillow, 2011). In this way, a person’s experience of vulnerability may change as a function of the relational context(s) that are present (Jordan, 2008).

Behaviorally, vulnerable behaviors are those that have historically been punished in social situations (Cordova and Scott, 2001). According to this perspective, what behaviors, topographically speaking, are vulnerable (i.e., what behaviors have been punished) vary between individual learning histories interacting with cultural norms. Extending from behavioral to a contextual behavioral perspective, Kanter et al. (2020) further characterize this class of previously interpersonally punished behaviors as including self-disclosure, emotional expressiveness, and emotional responsiveness. In other words, contextual behavioral explorations of vulnerability consider the effects of sharing personal information, communicating emotional state, and shifting verbal and affective communication to respond to another’s emotional state. This line of research positions vulnerability as a feature of relational closeness (Aron et al., 1997), emotional regulation (Panayiotou et al., 2019), relational aggression (Shea and Coyne, 2017), anxiety sensitivity associated with posttraumatic stress disorder (Bardeen et al., 2015), and more. The centrality of vulnerability to important outcomes has further supported its role in interventions designed to directly train intimacy (e.g., see Kanter et al., 2020).

## 3. Vulnerability-based intimacy interventions

Interventions have been developed to improve intimacy, but traditionally with a fairly narrow scope. Specifically, most have targeted persons in romantic relationships (see Kardan-Souraki et al., 2016 for a review of interventions to increase marital intimacy). Contextual behavioral interventions designed to promote intimacy (i.e., Functional Analytic Psychotherapy; FAP) have aimed for a broader scope. FAP involves directly training functionally vulnerable interactions, in which emotional expressions (i.e., emotional expressiveness combined with self-disclosure and invitations to self-disclose) evoke and reinforce emotional responsivity, and vice versa (Kanter et al., 2020). In this way, contextual behavioral interventions for building intimacy emphasize *interlocking behavioral contingencies* (IBCs; Glenn, 2004), in which one person’s behavior is functionally related to (i.e., sets the context for) another person’s behavior. These interventions also allow for consideration of cultural norms in terms of *metacontingencies* (Glenn, 2004), or the aspects of context that select for particular IBCs across groups.

Functional Analytic Psychotherapy (FAP; Kohlenberg and Tsai, 1991; Holman et al., 2017; Tsai et al., 2019) is a talk therapy approach wherein therapists address a client’s presenting problems by intervening on client’s in-session clinically relevant behaviors (CRBs) to enhance the client’s intimate relationships. Put another way, therapists working from a FAP perspective work to evoke and reinforce vulnerable interactions with their clients (CRB2s) as alternatives to the behaviors contributing to their difficulties (CRB1s). Systematic reviews investigating the effectiveness of FAP (e.g., Kanter et al., 2017; Singh and O’Brien, 2018) call for additional research with improved rigor, but emphasize that techniques and identified mechanisms of change (i.e., shifts in CRBs) are well supported when considering the therapist-as-social-reinforcer functions of FAP.

FAP has been proposed as particularly appropriate for establishing a therapeutic relationship in contexts where clients are likely to have punishing interpersonal histories, making these clients inherently more vulnerable (e.g., racially diverse client-therapist dyads, Miller et al., 2015; people struggling with gender and sexual minority stress, Skinta et al., 2018; transcultural or culturally sensitive services, Vandenberghe, 2008; Vandenberghe et al., 2010). FAP has also been extended beyond the psychotherapy context to training emotional rapport and responsiveness in ways that significantly improve medical doctors’ interactions with Black patients (Kanter et al., 2020). Finally, FAP has been applied in groups to promote intimacy (i.e., connectedness) in college students across racial differences (Kanter et al., 2019) with promising results, particularly for white participants (Williams et al., 2020). One topic of particular importance in vulnerability-based intimacy interventions, especially as they are extended to benefit inherently vulnerable interactions outside of the therapy context, is safety.

Kanter et al. (2020) describe safety as foundational to emotional responsiveness. The authors described promoting *safety* functionally as “engaging in non-verbal and verbal responses that decrease a speaker’s perceptions of threat and emotional arousal when engaged in non-verbal vulnerable emotional expressions” (p. 79). Kanter et al. (2020) further specify three formal categories of safety-providing responses: (1) synchronized emotional expressiveness, (2) indicators of interest, care, and affiliative intent, and (3) reciprocal vulnerable self-disclosures. This model acknowledges that these responses are “functionally complex” (i.e., have multiple functions), but that safety functions are imperative (Kanter et al., 2020). Whether such “safety-providing responses” function to decrease threat and nervous system activation to foster intimacy may require further conceptualization of the range of complex functions vulnerability can take on.

## 4. Re-considering functions of vulnerability

The vulnerability of behavior in a particular context has been functionally defined using its historical consequences (i.e., previously interpersonally punished; Cordova and Scott, 2001). Similarly, the intimacy of an interaction in a particular context is functionally defined in terms of both historical and immediate consequences (i.e., previously interpersonally punished, currently interpersonally reinforced; Cordova and Scott, 2001). No distinction has been made, however, between the overarching effects of different types of immediate reinforcement (i.e., positive or negative reinforcement) and corresponding antecedents (i.e., motivating operations and discriminative stimuli) involved in the IBCs that comprise a vulnerable interaction. In particular, it may be that vulnerability can emerge in appetitive or aversive functional relationships with a context, the distinction having important practical implications for facilitating intimacy in applied contexts.

### 4.1. Aversive vs. appetitive functional relations

Punishment and negative reinforcement both involve behavior interacting with *aversive* events, or situations that the organism will work to avoid or escape (see Hineline, 1984; Hineline and Rosales-Ruiz, 2013). *Punishment* is a process in which a behavior decreases in probability or frequency due to contact with aversive contexts, and *negative reinforcement* is a process in which behavior increases due to decreased contact with aversive contexts. In other words, punishment and negative reinforcement contingencies can be collectively described as involving *aversive control*, or, more broadly speaking, aversive functional relations between behavior and context.

*Aversive functional relations* are characterized by a narrowing of the entire contingency, or the field of factors comprising the interaction between behavior and context [e.g., conditioned suppression, Lyon (1968)]. Aversive functional relations thus involve a narrowing of context, where those stimuli available and accessible (i.e., to serve eliciting, evocative, discriminative, and/or consequential functions) are limited to aversive events and events that predict their reduction or absence. Aversive functional relations also involve a narrowing of behavior, where the available repertoire is limited to those operant behaviors involved in escape or avoidance and the co-occurring elicited subtle behaviors (e.g., Lovibond, 1970). The relative constriction of ongoing aversive functional relations between context and behavior results in an insensitivity to shifts in context (Ramnerö et al., 2015), thereby making aversive functions particularly persistent (e.g., Hoffman et al., 1966). The cumulative effect of aversive learning is increased sensitivity to aversive contexts and, in turn, an increasingly narrow and rigid repertoire (Hineline, 1984; Ramnerö et al., 2015).

Positive reinforcement, on the other hand, involves behavior interacting with *appetitive* events, or those that the organism will work to access. Indeed, *positive reinforcement* is a process in which a behavior increases in probability or frequency due to resulting increased contact with appetitive contexts. As such, positive reinforcement contingencies can be described as involving *appetitive control*, or, more broadly speaking, appetitive functional relations between behavior and the contexts, antecedent and consequential, in which that behavior occurs.

*Appetitive functional relations* are characterized by a broadening of the entire contingency, or the field of factors comprising the interaction between behavior and context (Wilson and DuFrene, 2009). Appetitive functional relations thus involve a broadening of context, where those stimuli available and accessible to serve eliciting, evocative, discriminative, and/or consequential functions are expansive and flexible. Access to a broader range of events that may function as context comes with a broader range of accessible behaviors, including operant behaviors generally involving seeking, exploring, and engaging, and the co-occurring elicited subtle behaviors. The relative breadth and flexibility of ongoing appetitive functional relations between context and behavior results in sensitivity to shifts in context (Skinner, 1958). In this way, appetitive functional relations are associated with increased *degrees of freedom* (i.e., alternative accessible behaviors; Goldiamond, 1975, 1976), and the subjective experience of choice. In contrast with aversive functional relationships, the cumulative effect of appetitive learning is increased sensitivity to appetitive contexts, and, in turn, an increasingly broad and flexible repertoire (Louisiana Contextual Science Research Group, 2022).

### 4.2. Intimacy involves vulnerability under appetitive functional relations

Vulnerability is central to intimacy, but it may not be a sufficient condition for intimacy to emerge. Instead, the current conceptualization suggests that intimacy requires that vulnerable behaviors (i.e., self-disclosure, emotional expressiveness, and emotional responsiveness), despite a history of being met with aversive consequences, emerge under appetitive functional relations with the context. Appetitive functional relations are observable in both operant form of vulnerability (where behaviors are shaped by a broad range of appetitive consequences and the evocative and discriminative contexts associated with them), and respondent forms of vulnerability (where emotions and their neurological correlates naturally and easily co-vary with the changing interpersonal context). Consequently, the vulnerability repertoire that contributes to intimacy emerges as broad, flexible, and sensitive to expansive appetitive learning experiences and continual adaptation to new interpersonal connections.

Unfortunately, not all contexts that foster vulnerability are appetitive. The present conceptualization of vulnerability also suggests that self-disclosure, emotional expressiveness, and emotional responsiveness can emerge in aversive functional relations. In fact, because vulnerable behaviors have, by definition, been historically met with aversive consequences, contexts where vulnerability is available (i.e., situations that are emotionally evocative) necessarily have some aversive functions. Kanter et al. (2020) note the salience of aversives in vulnerable interactions in their discussion of safety, emphasizing that safety-providing behaviors reduce threat and nervous system activation. This conceptualization suggests the importance that safety (i.e., the reduction of threat and activation) be offered as an antecedent for vulnerable behavior, rather than a consequence. To the extent that vulnerability is consequated with reduced contact with aversives (i.e., via negative reinforcement), vulnerability becomes more probable, but the functional relations at play are aversive. Aversive functional relations are observable in both operant aspects of vulnerability (where behaviors are shaped by a narrow range of aversive consequences and their antecedent evocative and discriminative contexts), and respondent aspects of vulnerability (where emotions and their neurological correlates diverge). Thus, the vulnerability repertoire that prevents intimacy emerges as narrow, rigid, insensitive to learning experiences outside of those that foster quicker or more effective avoidance and overgeneralized to any emotionally evocative interpersonal situation.

Certain contexts may include aversive functional relations that call for vulnerability, but vary in the extent to appetitive antecedents and consequences promote intimacy and subsequent well-being. For example, a student may recognize the need for accommodations in a course taught by a new professor, which would require an uncomfortable disclosure of their medical or psychological history. If the professor has not made explicit what accommodations may be available, how they can be accessed, or how they influence learning, the student may be forced to either initiate a vulnerable exchange without the safety of intimacy or simply proceed without the needed accommodations. Conversely, the professor could pre-emptively describe certain easily accessible accommodations as part of the learning environment with clear instructions on how to access them, how to know that they are needed, and how learning outcomes might be impacted by. In doing so, the context, despite having some aversive aspects for some inherently vulnerable students, is now better organized to foster appetitive functional relations with the vulnerable behavior involved in accessing needed accommodations. This allows not only for appetitives available in the intimate exchange, but also access to broader appetitives available in the course.

### 4.3. Intimacy involves vulnerability with consent

Considerations of functional relations in terms of their appetitiveness and aversiveness bring to bear a behavioral conceptualization of freedom vs. coercion. Skinner (1971) stated that freedom was defined by (1) the absence of aversive control via negative reinforcement or punishment, and (2) the absence of control via immediate positive reinforcement with deferred long-term aversive consequences. Freedom has also been related to the possibility or availability of choice, either choice of response options (Baum, 2017) or choices of alternative conditions (Catania, 1980). Similarly, *coercion* has been defined as control mediated by threats of punishment (Sidman, 1989, 1993), limited availability of choices (Goldiamond, 1975, 1976; Catania, 1980), and reduced access to resources needed to generate responses (Goltz, 2020). Said functionally, appetitive functional relations are associated with *genuine* choices and more *degrees of freedom* by Goldiamond (1975, 1976) – greater the sensitivity to various contexts (antecedents and consequences), greater the alternative accessible behaviors, greater freedom associated with the behavioral repertoire. Likewise, aversive functional relations are associated with limited options and greater *degrees of coercion* (Goldiamond, 1976). According to this conceptualization, contexts that foster vulnerability will only foster intimacy to the extent they maximize degrees of freedom and minimize degrees of coercion.

Kanter et al. (2020) approach this issue by specifying *asking*-*giving relations* as part of their model of intimacy. In this model, *asking* involves requests by the speaker for relational and/or non-relational needs to be met, and *giving* involves responding to the specific needs of the speaker by the listener. The authors discuss the risks inherent in the asking-giving interaction for both the speaker engaging in a vulnerable disclosure, and the listener accurately and empathically responding with emotional validation for such disclosures. For example, people asking may fear that their expression will result in conflict, rejection, or threats to their autonomy. This heightens the aversive functional relations involved in their vulnerable behavior. Furthermore, individuals giving may respond to the speaker’s requests inaccurately, insufficiently, or excessively. Therefore, asking behaviors may function aversively for the speaker. In line with the current conceptualization, the more that aversive functions dominate asking and giving at the individual level, the more likely they are to dominate the IBCs involved in the interaction.

The asking-giving exchange can be extended functionally by considering the negotiation of consent between interacting individuals. Consent is a complex interpersonal phenomenon with ethical implications in a range of contexts (Miller and Wertheimer, 2010). *Affirmative consent*, involving asking for and earning enthusiastic approval for an interaction, was first introduced in the context of sexual interactions (see Mettler, 2018) and is increasingly applied in functionally similar interactions (e.g., online interactions on social media; Im et al., 2021). Behaviorally, affirmative consent is an appetitive functional response class that (1) allows for the interacting people to *tact* (i.e., a verbal response evoked by an event or aspect of an event; Skinner, 1957) appetitive contingencies for themselves and each other, (2) allows for the interacting people to *mand* (i.e., a verbal response reinforced by a characteristic consequence associated with setting events; Skinner, 1957) for others to do the same, and (3) expands the degrees of freedom for the interacting behavioral repertoires with an ongoing availability of genuine choices that are responsive to shifting contingencies (Louisiana Contextual Science Research Group, 2021). In extension, this conceptualization would suggest that vulnerability fosters intimacy not only to the extent that that vulnerability is under appetitive functional relations, but also to the extent that an affirmative consent process has taken place. In other words, to foster intimacy, asking and giving should involve the naming of and responding to needs (1) under appetitive functional relations, and (2) with specification of not only the aversive but also the appetitive contingencies involved in those needs. Both requesting *and* providing ongoing consent mitigate some of the risks of contacting aversives for all persons in the interaction and increase emotional closeness as consent signals shared values around safety and well-being, both of which are necessary to foster intimacy.

Certain contexts may include aversive aspects that call for vulnerability but vary in the extent to which they foster affirmative consent. For example, two queer therapists (A and B) are having lunch in their practice’s kitchen when one (Therapist A) brings up the topic of discrimination at their practice and in the profession broadly. Therapist Aspeaks with great emotion about their past experiences and fears about taking on queer trainees. They also offer to listen and to provide support if Therapist B has similar experiences to share. An affirmative consent process is likely to begin if Therapist A not only tacts the aversive contingencies involved in their present vulnerability (e.g., “I’m feeling really upset by an unpleasant interaction I had with the boss, particularly considering the pressure to hire more queer trainees next term!”) but also (1) tacts the appetitive contingencies (antecedents and consequences) present in this context (e.g., “I’d really like to share what happened and how I’m carrying it. I think I’m looking for a sort of gut check.”) and (2) effectively mands for the first therapist to do the same (e.g., “How are you hearing all this? Do you have the space to listen? Do you have something you’d like to use lunch today for instead?”). The consent process continues to the extent that Therapist B is able to offer the same tact-mand combination (e.g., “Whoa. I wasn’t actually prepared for all that. And I do not know that I’ve thought about my experiences through the lens that you are asking for. I think I’d like more time to process what you have shared already before we go any further. I’d love to schedule a time to revisit this when I’m not hungry and stressed. Could I also help brainstorm some other ways you could get some support around this? Does that feel ok?”). Such affirmative consent interactions might be even more important when vulnerability is being invited in relationships with apparent disparities in power, such as in challenging training activities, therapy exercises, or employee feedback sessions.

### 4.4. Intimacy involves vulnerability with empowerment

Relative aspects of interacting repertoires with respect to the availability and accessibility of appetitives may contribute to the likelihood of vulnerability being (1) under appetitive functional relations, and (2) functionally consensual, both of which may be necessary for fostering functional intimacy. Maximizing appetitive functional relations involved in IBCs necessarily involves addressing and mitigating barriers in access to appetitives, and thus, addressing and responding effectively to privilege and power.

#### 4.4.1. Privilege

A feminist understanding of *privilege* as an “unearned advantage.. [and].. conferred dominance” (McIntosh, 1988, p. 1) has enabled a prior contextual behavioral conceptualization of privilege as differential access to important reinforcers (Terry et al., 2010). A similar behavioral conceptualization expands upon this idea, describing privilege as a dynamic ratio of appetitives to aversives accessible in any given context (Louisiana Contextual Science Research Group, 2022). In this way, disparities in privilege can be understood in terms of relative access to appetitives proportional to aversives both in their learning history and brought to bear in the immediate context. Thus, the repertoire of a person with more relative privilege is more broad, flexible, sensitive to appetitives, and likely to enter appetitive functional relations with the context. In contrast, the repertoire of a person with less relative privilege is more narrow, rigid, sensitive to aversives, and likely to enter aversive functional relations with the context. For example, a Black woman serving as the dean of a college may experience microaggressions and tone-policing based on gendered and racial stereotypes (e.g., “angry Black women;” Walley-Jean, 2009) when delivering a call-to-action to a predominantly white faculty body following a publicized occurrence of police brutality and systemic racism. Despite her leadership position as the dean and the appetitives that that position makes available, a learning history involving intersecting dimensions of racism, sexism, and misogyny brings aversives to bear in the current context, including speaking in group meetings, crafting written statements, and even processing their personal emotional reaction to the tragedy. The same gendered and racialized stereotypes contribute to disparate performance evaluations and leadership assessments (see Motro et al., 2022) serving to further the aversive contextual functions that contribute to her lack of privilege in this context.

*Power* is a central theme in feminist theory defined in a number of ways, including as a resource, as domination of others (i.e., “power-over”), and as empowerment to foster change (i.e., “power-to”; Allen, 2005). Contextual behavioral conceptualizations of power have also varied along similar themes. For example, Baum (2005) defined power as “the control that each party in a relationship exerts over the other’s behavior” (p. 235). This access to control remains central to other proposed definitions of power (Guerin, 1994; Biglan, 1995). It has also been specified that this access to control is exerted relationally via control over a relatively greater number of significant reinforcers (Terry et al., 2010). Consistent with the contextual perspective on privilege, *power* has been conceptualized as the degrees of freedom afforded by access to appetitives and the resulting expansive repertoire (Louisiana Contextual Science Research Group, 2022). In this way, disparities in power can be understood in terms of relative degrees of freedom fostered by one’s relative privilege. More power involves greater degrees of freedom fostered by greater privilege and the associated ease of access to appetitives relative to aversives. Less power, on the other hand, involves fewer degrees of freedom fostered by less privilege and the associated dominance of aversives relative to a scarcity of available and accessible appetitives.

Thus, power is contextually-bound, where some contexts may function as *empowering* (i.e., fostering greater degrees of freedom via improved access to appetitives and buffering the impact of aversives) and others may be *disempowering* (foster reduced degrees of freedom via increased salience of aversives, and reduced access to appetitives). For example, a gender-marginalized faculty member working in a graduate school is at increased risk of interpersonal threats, ranging from microaggressions to overt harassment, and self-advocacy in these contexts may adversely impact their work experience and career trajectory (see Blithe and Elliott, 2020). Here, the graduate school could be described as a disempowering context for that faculty member.

Applied to interpersonal interactions, this conceptualization suggests that privilege and power are not static, finite resources allocated in an interaction according to persistent identities. Instead, privilege and power are dynamic, contextually-bound functional aspects of the stimulating context and the current repertoire, respectively. IBCs do not function in such a way as to empower one person (i.e., increasing access to appetitives and increasing degrees of freedom) *by* disempowering the other (i.e., increasing access to aversives and decreasing degrees of freedom). Rather, IBCs could emerge that empower all parties involved in a vulnerable interaction. In fact, this centering of appetitive functional relations that are mutually expansive may be exactly what is necessary for vulnerability to cultivate intimacy.

The less power and privilege a person has in an interpersonal interaction, the more likely they are to respond to invitations to vulnerability under aversive functional relations due to the relative dominance of aversive learning in their history in similar contexts. In other words, the more disempowering an interpersonal context is (i.e., the fewer degrees of freedom available there), the more likely invitations for vulnerability will function aversively, evoking more vulnerability or less, depending on which has historically allowed them to minimize contact with the aversive in similar contexts. The gender marginalized faculty member mentioned above will require more support (i.e., appetitives) in their vulnerable interpersonal interactions with other faculty to overcome the broadly disempowering context to connect intimately.

Promoting mutually appetitive vulnerable IBCs may be most challenging when power and privilege are disparate between people in an interpersonal interaction. Disparities in power and privilege involve disparities in the distribution of aversive vs. appetitive functional relations obtaining in any one moment and, thus, the relative likelihood of aversive vs. appetitive learning opportunities in that situation. Such disparities are problematic in several ways (see Louisiana Contextual Science Research Group, 2022), but perhaps most so in vulnerable interactions, where the probability of vulnerable behaviors occurring under appetitive functional relations can be significantly reduced despite best efforts. While disparities in privilege and power are unavoidable in most interpersonal interactions, introducing vulnerability to those interactions is likely to evoke behaviors that emerge from and maintain such disparities in power and privilege and prevent true intimacy (i.e., sociopolitical problematic behaviors, SP1s; Terry et al., 2010).

The effects of aversive functional relations around vulnerability vary depending on the person’s repertoire with such contexts. To the extent that the disempowered person’s lack of privilege and power are generalized across interpersonal contexts (e.g., with intersecting identities that limit power and privilege broadly), they are also more likely to have an explicit learning history about the emotions of more powerful people being aversive. For example, the phenomenon of *white tears*, where people of color are oppressed by the emotional expressions of white people, is well documented (Accapadi, 2007). Here, a less powerful person may learn to engage in vulnerability (i.e., self-disclosure, emotional expressiveness, and emotional responsiveness) as a way of calming the more powerful person, not in pursuit of connection or soothing for themselves, but as a way to escape a historically threatening interaction (see Menakem, 2017).

This dynamic would also be considered problematic when the more powerful and privileged person’s vulnerability is under aversive functional relations. This can occur due to some aversive aspect of context outside of the interpersonal interaction (e.g., an upsetting conflict with a family member, a stressful financial challenge, a frightening storm outside). This can also occur when a learning history where interacting with people with less power is aversive in and of itself, reducing degrees of freedom without equalizing the disparity. For example, some conceptualizations of racialized trauma highlight the pervasive socialization in the U.S. around Black bodies as impervious, dangerous, hypersexual, and dirty, along with the resulting physical, emotional, and mental constriction experienced in their presence (see Menakem, 2017). If the more powerful person’s current behavior is being dominated by aversives, the interaction is likely to be increasingly and rigidly focused on reducing their distress. In other words, if the emotional expressions and responsiveness of the person with relative ease of access to appetitives is still under aversive functional relations, those functional relations are likely to dominate the IBCs for both members of the interaction.

Consider the example of a professor serving as a thesis advisor arriving late for a meeting with their graduate student. The interaction may begin with the professor apologizing and explaining to the student that they had been fighting with their partner, which resulted in them leaving home late. As they are sharing this story, the professor offers some background as to why their conflict with their partner is so upsetting, becoming teary-eyed and expressing other overt signs of emotional distress. The professor is demonstrating vulnerability and may struggle to contact the empowering appetitives available in the thesis work, the mentoring relationship, or the pride in their professional position. Meanwhile the student is confronted with the professor’s vulnerability without the power and privilege that would allow them to contact their own empowering appetitives. For example, it is unlikely that the student would have the degrees of freedom, shaped by an appetitive learning history, to initiate a consent process in which they could name their desire to return to the meeting’s original agenda (their thesis), their need for support around that work, and their preference to reschedule the meeting if their professor cannot meet that need. The student’s learning history may also involve specific aversive consequences for engaging in such behaviors such as acute punishing feedback or longer-term damage to the relationship. So instead, the student is likely to find themselves trying to calm their professor to allow them relief.

Such aversive functional relations around vulnerability could also arise with the more empowered and privileged person inviting vulnerability. For example, a therapy trainee finds themselves in a clinical supervision meeting, being asked by their supervisor to share their painful feelings, self-deprecating thoughts, and patterns of unworkable action. The supervisor is alarmed by the trainee’s rigidity and wants to offer them an opportunity to build their repertoire before it negatively impacts their therapy work. The therapy trainee is aware of their suffering and how important their personal growth could be to their professional development but finds themselves feeling overwhelmed by their supervisor’s softened tone and intense eye contact. The therapy trainee may additionally experience concerns about their vulnerabilities (e.g., painful feelings, self-deprecating thoughts, unworkable actions) being used against them in formal evaluations. In this way, both the supervisor’s and the trainee’s repertoires are dominated by aversive functional relations. The disparity in power and privilege further limits the trainee’s capacity to object to the line of questioning. The trainee discloses as requested by the supervisor but leaves the meeting confused about what the purpose of their disclosure was and how to move forward with their therapy sessions. On the one hand, the trainee feels heard and accepted by their supervisor, but on the other hand, they are primarily dreading having their personal psychological struggles present in their future meetings. Further, this dread may be founded, as the supervisor experienced relief at the trainee’s openness and their probing was reinforced.

## 5. Elaborated contextual behavioral conceptualization of intimacy

This conceptualization builds on existing behavioral and contextual behavioral approaches to understanding and intervening on intimacy. From this perspective, intimacy involves vulnerable behaviors, or responses to aversive antecedents, that are under appetitive functional relations, consensual, and empowered (see Table 1). To this end, contexts that aim to intervene to increase intimacy will, in the presence of aversive antecedents: (1) evoke behavior that functions to increase ongoing contact with shifting appetitives, (2) involve tact-mand combinations that make appetitive contingencies salient, and (3) include behaviors that support increasing degrees of freedom across IBCs. Implications for creating contexts for intimacy vary across power and privilege disparities, relational goals, and time points within the interaction.

**Table 1:** Conditions necessary for functional intimacy.

Intimacy involves…	Relevant terms
Vulnerability	*Vulnerability*–socially risky behavior; behavior that has been previously punished; has aversive antecedents
Vulnerability under appetitive functional relations	*Appetitive functional relations*–seeking, exploring, and engaging behaviors; broad and flexible repertoire, broad and flexible context; subjective experience of choice; strengthening of appetitive learning vs. *Aversive functional relations*–running, fighting, and hiding behaviors; narrow and rigid repertoire, narrow and rigid context; subjective experience of coercion; strengthening of aversive learning
Vulnerability with consent	*Affirmative consent*–requesting and receiving enthusiastic approval for an interaction; appetitive functional response class involving a tact-mand combination; interacting people tact appetitive contingencies at play and mand for others to do the same; expands degrees of freedom with an ongoing, shifting availability of genuine choices vs. *Coercion*–interaction persists with absent, limited, or threatening communication; aversive functional response class focused on promoting the interaction with little attention to current function; if tacted at all, appetitives are presented in ways that suggest scarcity or otherwise, narrow degrees of freedom
Vulnerability with empowerment	*Empowerment*–positions one with the power to act to resource current needs; fosters greater degrees of freedom via improved access to appetitives and buffering the impact of aversives vs. *Disempowerment*–positions one to act to resource the needs of the more powerful person; fosters reduced degrees of freedom via increased salience of aversives, and reduced access to appetitives

*Functionally intimate contexts will, in the presence of aversive antecedents: (1) evoke behavior that functions to increase ongoing contact with shifting appetitives, (2) involve tact-mand combinations that make appetitive contingencies salient, and (3) include behaviors that support increasing degrees of freedom across IBCs.*

### 5.1. Creating contexts for intimacy

**Figure 1:**
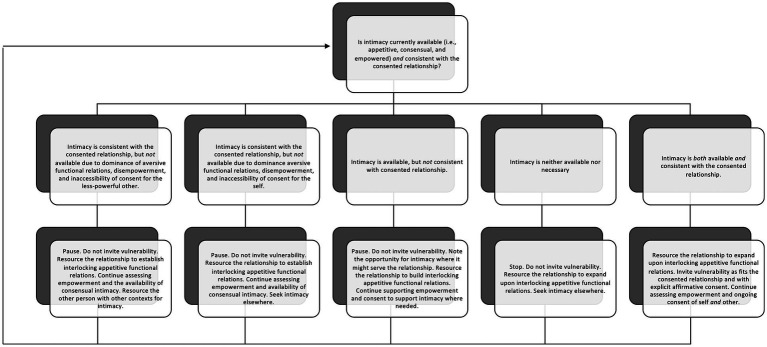
Ongoing assessment of conditions for functional intimacy.

According to this conceptualization, the person with more relative power and privilege in an interaction and overall relationship bears responsibility for managing vulnerability in such a way as to promote intimacy instead of coerced vulnerability. A person with more relative power and privilege is likely to be more sensitized to available appetitives and have appetitive learning as a more robust aspect of their repertoire. Sensitivity to appetitives is necessary for consensual interactions and empowerment, both being critical features of training or intervening on intimacy. In this way, the reader is invited to reflect upon the relational contexts in which their ratio of appetitive to aversive functional relations (i.e., privilege) maximizes their degrees of freedom (i.e., power), and to consider the following recommendations for fostering appetitive functional relations when aversives (i.e., conditions for vulnerability) are present. Figure 1 offers a process for moment-to-moment assessment of conditions for functional intimacy along with response options based on observed conditions.

#### 5.1.1. Modulate mands for intimacy according to relative power, the consented relationship, the consented purpose of the interaction, and other aspects of the immediate context

Disparities in power and privilege will always be present in relationships and will fluctuate across the different contexts in which relating occurs. Further, relationships, both personal and professional, come with distinct responsibilities that may or may not involve intimacy. For example, a Psychology professor’s responsibilities to their students differ from those to their applied supervisees, which differ still from those to their clients in their therapy or consultation work. While intimacy is central to well-being, this conceptualization suggests that intimacy is simply not always available or necessary, and vulnerability should only be invited where it is. The responsibility for determining if intimacy is available in a particular relationship and context fall to the person with more power, and involve moment-to-moment assessment of: (1) power dynamics as the relative ratio of appetitives to aversives available for each member of the interaction, (2) consistency of intimacy with the consented relationship p and the purpose of the current interaction in terms of explicitly tacted interlocking appetitives, and (3) other aspects of the immediate context in terms of their appetitiveness or aversiveness. We might ask: How much breadth, flexibility, and freedom do they seem to have in this interaction? How much breadth, flexibility, and freedom do I have? What are we each working for here? Is there anything we seem to be working to minimize, delay, or escape? Is there anything we seem to be grasping? Is building our intimacy an aspect of why we are in a relationship with one another? Is building our intimacy an aspect of why we are interacting right now? What seems to be supporting or limiting my freedom? What seems to be supporting or limiting their freedom? When this assessment suggests that the context offers limited support for intimacy – that is, that either the relationship or extrarelational aspects of context are limiting empowerment, consent, or overall access to appetitives, invitations to vulnerability should be tempered or withdrawn. When this assessment suggests that the context supports intimacy – that is, that the relationship is appetitive, empowering, and consensual, invitations to vulnerability have the potential to foster intimacy.

#### 5.1.2. Foster accessibility of appetitives in terms of the detection, discrimination, and tacting appetitives with contextually appropriate resourcing

Sometimes the consented relationship and purpose of the interaction does involve intimacy – that is, sometimes intimacy is an explicitly-tacted appetitive process or outcome for the relationship broadly and the current interaction. For example, a psychotherapy relationship is, by definition, intimate. However, just because intimacy is a consented part of the relationship and the interaction does not mean it is available from its initiation. For example, a psychotherapist may have to put significant effort into establishing the context for vulnerability to foster intimacy. Repertoires involved in accessing appetitives (both relational and otherwise) vary considerably between people and contexts, and are challenged by vulnerability. Thus, the person with more power in the consented intimate relationship and current interaction bears responsibility for fostering the accessibility of appetitives for the person with less power, both prior to and during the introduction of aversives involved in vulnerability intended to promote intimacy. This involves creating a context that evokes and reinforces the detection of appetitive functional relations, the discrimination of behavior necessary to access them, and the tacting of shifting appetitive functions as the interaction unfolds. The psychotherapist working from this perspective might invite the client to contact appetitives in their interaction from the most simple (e.g., inviting the client to give themselves a kind and resourcing breath) to fairly complex (e.g., inviting the client to share what life is or has been like when they were not struggling or to react to the therapist’s shared intentions for psychotherapy). Here, the psychotherapist bases their invitations on the client’s responses, interacting to support the gradual cultivation of this skill of detecting, discriminating, and tacting appetitive contingencies in the therapy interaction. It is also necessary for the more powerful person to determine if the current context can be appropriately resourced with appetitives salient to the person with less power to foster accessibility for them and promote intimacy in the relationship. If a psychotherapy client struggles to interact appetitively in a particular session, in the therapy relationship, or at all, the vulnerability of the interaction might already be outstripping the appetitives available, and the appetitive learning repertoire and/or the therapeutic relationship may need to be developed before vulnerability is explicitly invited.

#### 5.1.3. Foster one’s own access to appetitives in terms of the detection, discrimination, and tacting appetitives with contextually appropriate resourcing

As vulnerability necessarily involves contact with aversives, contexts that evoke vulnerable behavior can disempower even people with more relative power in the interaction. To the extent that the more powerful person continues to engage vulnerably, then, their behavior is likely to come under aversive functional relations. In other words, their behavior is likely to narrow and become increasingly rigid, reducing their sensitivity and responsiveness to the varying context created by the less powerful person’s behavior. In this way, individual-level aversive functional relations are likely to extend into IBCs and further limit access to appetitives for all members with varying degrees of power in the interaction. Thus, the person with more power in the interaction is responsible for fostering ongoing accessibility of appetitives not only for those with less power, but also for themselves. This involves assessing one’s own current repertoire for the detection, discrimination, and tacting of appetitives both prior to and during the interaction. Prior to initiating a vulnerable interaction, the person with more power may find themselves rigid, narrow, and highly oriented to aversives (i.e., aversive functional relations may be dominant in their repertoire). For example, a highly paid keynote presenter who is jet lagged, hungry, and dehydrated may find themselves struggling to connect fully with an emotionally compelling personal story they planned to use to introduce their talk. Despite them being positioned to have the most power and degrees of freedom at the event, they simply do not have the resources to interact with the memories, the images on slides, and the audience appetitively. They rehearse the opening again and again, noticing themselves feeling more and more distracted, anxious, and disconnected from the memory with each attempt. If they are not able to engage appetitively with this vulnerable expression, it is highly unlikely that any of their audience members will. Here, the vulnerability is likely to be alienating instead of connecting. Such an effect might be even more pronounced if the audience is small and intended to be intimate.

In such a situation, intimacy is unavailable and should not be pursued. Instead, the person with more power may focus temporarily on resourcing themselves (i.e., engaging in behavior to increase salience of and contact with appetitives) to reassess the importance, availability, and necessity of intimacy. This might include actions that vary in complexity, including those that address physiological needs (e.g., resting, breathing deeply and mindfully, eating or drinking, exercising), interpersonal needs (e.g., seeking consultation, validation, or support from a similarly powered peer), or intrapersonal needs (e.g., reflecting on relevant values, affirming relevant aspects of identity). If there is sufficient time, instead of rehearsal, the talk might be better served by resourcing the speaker themselves with some fluids, a snack, and a nap. If time is limited, the speaker might replace the story with one that is less personal or emotionally compelling that they can interact with effectively. Notably, accessibility of appetitive functional relations for the person with more power is necessary *and insufficient* for intimacy. If self-assessment reveals increased breadth and flexibility and corresponding access to appetitives, they may initiate the intimate interaction, with careful attention to ongoing accessibility of appetitive functional relations for themselves and other members of the interaction, and a commitment to resource themselves as needed to initiate, maintain, or withdraw intimacy.

#### 5.1.4. Assess for increasing prominence of aversives in the ongoing intimate interaction and intervene to maintain the dominance of appetitive functional relations

Interactions are a dynamic process (as are consent and power), and an interaction can shift at any point in such a way as to increase the prominence of aversive functional relations, increasing the likelihood of disempowerment and withdrawal of consent. For example, a parent may invite their adolescent child to talk about a long-term friendship that seems to be preoccupying them. The child accepts, disclosing a number of quite dangerous things their friend has been doing. As the parent’s concern increases, they may notice the child becoming defensive and sounding like they might try to end the conversation. The responsibility for ongoing assessment of aversives associated with vulnerability falls to the person with more power, as does the responsibility for shifting the context to maintain the dominance of appetitive functional relations. This involves watching other members of the interaction for narrowing or increased rigidity of their repertoire, and resourcing them as needed (i.e., shifting the context as needed to increase salience of and contact with appetitives). This might begin with inviting actions that address physiological needs (e.g., inviting resting, breathing deeply and mindfully, eating or drinking, exercising) and extend into interpersonal needs (e.g., inviting validation-seeking, or support from a similarly powered peer), or intrapersonal needs (e.g., inviting reflection on relevant values, affirming relevant aspects of identity). For example, the parent may express gratitude for the disclosure and invite their child to pause to see if they need a hug or a glass of water before they go on. If ongoing assessment reveals increased breadth and flexibility and corresponding access to appetitives, they may re-initiate the intimate interaction by inviting vulnerability (i.e., re-introducing aversives), with careful attention to ongoing accessibility of appetitive functional relations. For example, the parent might acknowledge how hard it must have been to keep these secrets about someone they care so much for, and invite them to discuss it further. The parent might also stop short of sharing their own concerns for the friend, noticing that their child does not seem well-positioned to respond appetitively to their expression. Ideally the increased prominence of aversive functional relations is detected prior to their dominance, allowing for a shift in functional relations to maintain the dominance of appetitive functional relations before the person engages in avoidance behavior. Otherwise, intimacy may become unavailable for the remainder of the interaction, as the capacity to contact aversives associated with vulnerability without them becoming dominant is limited.

#### 5.1.5. Establish interlocking appetitive functional relations

Many relationships call for some degree of consented intimacy (e.g., coworkers, neighbors, colleagues, community members, etc.), even if that is not the primary purpose of the relationship. In the context of such relationships, interactions that are not particularly vulnerable can provide a foundation for future intimacy through the development of robust and easily accessible interlocking appetitive functional relations (i.e., appetitive IBCs). Members of the interaction can learn early on in a relationship how to behave in ways that are mutually appetitive. In other words, people can learn to engage in behaviors that are under appetitive functional relations with the behaviors of the other, where each is evoking and reinforcing others’ behavior appetitively. Even if there is no apparent power differential associated with respective roles in the relationship, power will still vary across contexts and interaction dynamics. Thus, here too, fostering interlocking appetitive functional relations is the responsibility of the person with more power in the interaction. This involves the person with more power introducing appetitives that evoke and reinforce a range of behaviors for the person with less power, increasing the breadth and flexibility of their unfolding behavioral stream and sensitizing them to a range of appetitives in the context. Once the relationship is established to serve appetitive functions, the person with more power can specifically evoke and reinforce behaviors to support their own repertoire of assessing and intervening on appetitive functional relations (i.e., to evoke and reinforce improved sensitivity and responsiveness of the person with more power to the behavior of the person with less power). If possible, vulnerability should not be introduced until these interpersonal appetitives are well-established and easily accessible to both members of the relationship, in order to optimize the likelihood of intimacy. If not possible, vulnerable interactions should be resourced as described above and interspersed with interactions that are more exclusively appetitive, so as to continue to establish interpersonal appetitives to the point that intimacy is more readily accessible.

#### 5.1.6. Aversive consequences are not used to train behavior and aversive antecedents are limited to the consented relationship

In any relationship, there are behaviors in both people’s repertoire that are aversive to the other. In other words, there are behaviors in one person’s repertoire that narrow and rigidify the behavior of the other, and motivate the other person toward running, fighting, or hiding to decrease that behavior of the other. The quickest, most acutely effective way for a person to decrease a behavior of another is to punish it by presenting aversive consequences. For example, if a research assistant is dominating conversations in lab meetings, the quickest and most effective way to stop it would be to consequate it with an aversive. The research supervisor might, having seen how the assistant seeks the other members’ approval, admonish them firmly and publicly, watching them grow red with shame and withdraw. Regardless of whether the dominating behavior was under appetitive or aversive functional relations prior to the admonishment, aversive functions will now be prominent in lab meeting–for that assistant, and perhaps also for the supervisor and even other lab members on the team.

Similarly, the quickest, most acutely effective way to increase alternative, more desired behaviors is to reinforce them by removing aversive consequences. For example, a supervisor may lead their team in a way that is quite intimidating, offering only intermittent praise for employee work and frequently demonstrating high level skills with fluency instead of offering instruction or for how to reach that level. Here, the supervisor’s intermittent praise for technical improvements may actually function not as an appetitive, but as a temporary removal of threat, allowing the praised team member some relief from the constant intimidation. Aversive functions, however, will still be most prominent, as that relief is temporary, and technical improvements will be overly focused on supervisor reactions. Indeed the quickness and acute effectiveness of aversive functional relations to change another’s behavior allows for the behaviors that serve aversive functions to emerge quite easily and with the characteristic narrowness and rigidity. In short, the use of aversive functional relations by the person with more power, even when used with intention and care, fosters their dominance in the IBCs. Further, the quickness and acute effectiveness of behavior change via aversive functional relations is accompanied by serious costs to the availability of intimacy. To the extent that a person uses the application and removal of aversive consequences to train desired repertoires, they are increasing the likelihood that their presence will function aversively for the other person such that aversive functional relations easily become prominent in IBCs. This risk is elevated in vulnerable interactions, which necessarily involve the presence of aversives, and further elevated when power disparities emerge. Here, the use of aversive consequences is associated with decreased availability of appetitive consent and increased probability of disempowerment, both of which are necessary for intimacy. In both examples above, the researcher and the supervisor might find their teams not only struggling to grow expansively and flexibly, but also failing to meet the kinds of vulnerabilities that show up in any workgroup (e.g., errors, interpersonal conflicts, etc.) with intimacy.

Thus, according to this conceptualization, it is the responsibility of the person with more power in a particular interaction in any relationship to approach behavior change in terms of expanding repertoires under appetitive functional relations. When the person with less power engages in behavior that is aversive to the person with more power, the role of the person with more power is to evoke and reinforce a range of new, more effective behaviors. When aversives are introduced by a person with more power, they are preceded by foundational interpersonal appetitives and presented as antecedents to expand repertoires in vulnerable situations, instead of as consequences to limit the behavior they find aversive. In both situations above, the researcher and the supervisor’s teams would be well served by discussions to establish appetitives in the work, to nurture interpersonal appetitives, to develop appetitive functional relations in team processes, and to support self-resourcing with appetitives outside of the work.

## 6. Conclusion

Intimacy is considered integral to personal and relational well-being (see Reis, 1990), and, in most models, involves bidirectional vulnerability (see Gaia, 2002; Timmerman, 2009). FAP (Kanter et al., 2020) has provided a strong contextual behavioral foundation for the ongoing development of intimacy-based interventions, emphasizing contexts that foster vulnerability, intimacy, and safety. This conceptualization expands on that foundation suggesting that safe intimacy is only possible in contexts that use appetitive functional relations to promote consensual, empowered vulnerability. Most crucially, this conceptualization of intimacy places responsibility on people with more relative power to create appetitive contexts for intimacy and to avoid vulnerability where intimacy is not possible. It is our hope that this conceptualization may both guide intentional responses to the needs that vulnerable interactions present, and inform future empirical and applied developments in the science of intimacy in research, practice, and community culture.

## Author’s note

Because the authors (1) believe our respective absolute contributions were a function of disparities in resources and associated learning histories (i.e., our privilege) and (2) believe them to be functionally equivalent and indeed, inseparable, when considering interlocking contingencies, all authors have agreed to publish the paper in the name of our lab, each functioning as a representative thereof.

## Author contributions

All authors have made substantial contributions to the conceptual analysis offered, participated in drafting or revising the manuscript for submission, approved the final submitted version, agreed to be accountable for all aspects of the work, and agreed, in the name of the Louisiana Contextual Science Research Group (LCSRG), to the submission of this manuscript in this form.

## Group members of Louisiana Contextual Science Research Group

Jade Campbell, Jessica Criddle, LaGriff Griffin, Eva Lieberman, Michael May, Melissa Miller, Nicole Pyke, MaKensey Sanders, Emily Sandoz, Thomas Sease, Janani Vaidya, Jon-Patric Veal, Abbey Warren, (University of Louisiana at Lafayette, Lafayette, LA, United States).

## Funding

This work was partially funded by the Louisiana Board of Regents via the Emma Louise LeBlanc Burguieres/BORSF Endowed Professorship of Social Sciences and by a 2023 University of Louisiana at Lafayette Sustainable Development Research Grant.

## Conflict of interest

The authors declare that the research was conducted in the absence of any commercial or financial relationships that could be construed as a potential conflict of interest.

## Publisher’s note

All claims expressed in this article are solely those of the authors and do not necessarily represent those of their affiliated organizations, or those of the publisher, the editors and the reviewers. Any product that may be evaluated in this article, or claim that may be made by its manufacturer, is not guaranteed or endorsed by the publisher.
